# Renal dysfunction by baseline CD4 cell count in a cohort of adults starting antiretroviral treatment regardless of CD4 count in the HIV Prevention Trials Network 071 [HPTN 071; Population Effect of Antiretroviral Therapy to Reduce HIV Transmission (PopART)] study in South Africa

**DOI:** 10.1111/hiv.12729

**Published:** 2019-04-08

**Authors:** P Bock, K Nel, G Fatti, R Sloot, N Ford, J Voget, C Gunst, N Grobbelaar, F Louis, S Floyd, R Hayes, H Ayles, N Beyers, S Fidler, the HPTN 071 (PopART) Team

**Affiliations:** ^1^ Department of Paediatrics and Child Health Faculty of Medicine and Health Sciences Desmond Tutu TB Centre Stellenbosch University Cape Town South Africa; ^2^ City of Cape Town Health Services Cape Town South Africa; ^3^ Kheth’ Impilo, AIDS Free Living Cape Town South Africa; ^4^ Division of Epidemiology and Biostatistics Department of Global Health Faculty of Medicine and Health Sciences Stellenbosch University Cape Town South Africa; ^5^ Amsterdam Institute for Global Health and Development Amsterdam The Netherlands; ^6^ Centre for Infectious Disease Epidemiology and Research University of Cape Town Cape Town South Africa; ^7^ Western Cape Department of Health HIV/AIDS, STI & TB Directorate Cape Town South Africa; ^8^ Faculty of Medicine and Health Sciences Stellenbosch University Division of Family Medicine and Primary Health Care Stellenbosch University Cape Town South Africa; ^9^ Western Cape Department of Health Cape Winelands District Brewelskloof Hospital Worcester South Africa; ^10^ ANOVA Healthcare Paarl South Africa; ^11^ Independent Consultant Cape Town South Africa; ^12^ Department of Infectious Disease Epidemiology London School of Hygiene and Tropical Medicine London UK; ^13^ Department of Clinical Research London School of Hygiene and Tropical Medicine London UK; ^14^ Department of Medicine Imperial College London London UK

**Keywords:** antiretroviral treatment, CD4 count, HIV/AIDS, renal dysfunction

## Abstract

**Objectives:**

Renal dysfunction is a significant cause of morbidity and mortality among HIV‐positive individuals. This study evaluated renal dysfunction in a cohort of adults who started antiretroviral treatment (ART) regardless of CD4 count at three Department of Health (DOH) clinics included in the HIV Prevention Trials Network 071 (HPTN 071) Population Effect of Antiretroviral Therapy to Reduce HIV Transmission (PopART) trial.

**Methods:**

A retrospective cohort analysis of routine data for HIV‐positive individuals starting ART between January 2014 and November 2015 was completed. Incident renal dysfunction was defined as an estimated glomerular filtration rate (eEGFR) < 60 mL/min after ART initiation among individuals with a baseline (pre‐ART) eGFR ≥ 60 mL/min.

**Results:**

Overall, 2423 individuals, with a median baseline CD4 count of 328 cells/μL [interquartile range (IQR) 195–468 cells/μL], were included in the analysis. Forty‐seven individuals had a baseline eGFR < 60 mL/min. Among 1634 nonpregnant individuals started on a tenofovir‐containing ART regimen and with a baseline eGFR ≥ 60 mL/min, 27 developed an eGFR < 60 mL/min on ART. Regression analysis showed lower odds of baseline eGFR < 60 mL/min at baseline CD4 counts of > 500 cells/μL [adjusted odds ratio (aOR) 0.29; 95% confidence interval (CI) 0.11–0.80], 351–500 cells/μL (aOR 0.22; 95% CI 0.08–0.59) and 201–350 (aOR 0.48; 95% CI: 0.24–0.97) compared with baseline CD4 counts < 200 cells/μL.

**Conclusions:**

This study showed low rates of renal dysfunction at baseline and on ART, with lower rates of baseline renal dysfunction among individuals with baseline CD4 counts > 200 cells/μL. Strategies that use baseline characteristics, such as age, to identify individuals at high risk of renal dysfunction on ART for enhanced eGFR monitoring may be effective and should be the subject of future research.

## Introduction

The number of individuals on antiretroviral therapy (ART) continues to increase annually 1. Recent World Health Organization (WHO) recommendations to start ART regardless of CD4 cell count in high‐burden settings have been adopted by almost all countries and can be expected to lead to an increased number of HIV‐positive individuals starting ART at higher CD4 cell counts 2, 3.

Renal dysfunction is a well‐recognized cause of morbidity and mortality among HIV‐positive individuals with advanced disease and amongst those starting ART 4, 5. In HIV‐positive individuals, renal dysfunction may occur through various mechanisms, including HIV‐associated nephropathy (HIVAN), HIV immune complex kidney disease, thrombotic microangiopathy and toxicity from certain ART compounds such as tenofovir disoproxil fumarate (TDF) 4, 6. Although immune reconstitution after ART initiation is overall associated with improved renal function, for those starting therapy with advanced disease, ART initiation at HIV diagnosis irrespective of CD4 count may potentially have a negative impact on renal function through longer exposure to TDF toxicity 4, 5, 7, as TDF is a recommended component of first‐line ART globally 8. Routine monitoring of renal function in individuals starting TDF using the estimated serum glomerular filtration rate (eGFR) is desirable, and TDF is not recommended for individuals with eGFR < 60 mL/min at the time of ART initiation 9.

Programmatic studies from high‐burden settings show a varying prevalence of baseline renal dysfunction among individuals starting ART 10, 11, 12, 13, 14. The reported association between baseline CD4 count and baseline renal dysfunction also varies, with some studies showing higher baseline renal dysfunction at lower CD4 counts 12, 13 and other studies showing no association 10, 14.

An association has been reported between low CD4 count and renal dysfunction after ART initiation, from programmatic studies, with median baseline CD4 counts ranging from 154 to 209 cells/μL. These studies reported less renal dysfunction following ART initiation among individuals with baseline CD4 counts > 200 cells/μL compared with lower values 15, 16. Recently published results from the Strategic timing of antiretroviral treatment (START) trial showed higher mean eGFR after ART initiation among individuals starting ART at baseline CD4 counts > 500 cells/μL compared with those with baseline CD4 counts of 350–500 cells/μL 17. There are, however, limited data from programmatic studies to confirm these findings. This study aimed to evaluate the association between baseline CD4 count and renal dysfunction at baseline and after ART initiation among adults starting ART regardless of CD4 count at three Department of Health (DOH) clinics, included in the HPTN 071 [Population Effect of Antiretroviral Therapy to Reduce HIV Transmission (PopART)] trial, in South Africa.

## Methods

### Study setting

The HPTN 071 PopART study is a community randomized trial implemented in South Africa and Zambia. A full description of the PopART trial design has previously been published 18. For PopART, a community was defined as the catchment population of a DOH primary health care (PHC) clinic. PopART communities were randomly allocated to one of three arms: A, B or C. Arm A communities received the full intervention package, which included community HIV prevention services delivered by a cadre of community workers named Community HIV Care Providers (CHiPs) and ART regardless of CD4 count at the local DOH clinic. Arm B communities received the CHiP intervention with ART as per in‐country guidelines, while Arm C communities received standard care interventions only.

Two clinics were in an urban area and have been named ‘Metro 1’ and ‘Metro 2’; one clinic was in a rural area and has been named ‘Rural’. All three study clinics offered the same package of PHC services. ART services at the three study clinics were provided according to provincial DOH ART guidelines 19, with the exception that all three study clinics offered ART regardless of CD4 count from 1 January 2014, before the provincial DOH ART guidelines changed to ART regardless of CD4 count in October 2016, as defined in the trial protocol 19. As part of routine care, all individuals starting ART were recorded in the electronic HIV programme monitoring system, Tier.net 20. In accordance with South African guidelines, a fixed‐dose combination tablet of TDF, emtricitabine and efavirenz (TEE) was used as the first‐line ART regimen of choice. All laboratory services were provided by the centralized regional National Health Laboratory Services (NHLS) with daily pick‐up of specimens from all PHC clinics. TDF was contraindicated in individuals with baseline eGFR < 50 mL/min in whom zidovudine (ZDV), stavudine (d4T) and abacavir (ABC) were recommended first‐line alternatives 21. For individuals starting TDF, serum creatinine used for eGFR estimation was measured prior to ART initiation, at 1, 4 and 12 months after initiating therapy, and annually thereafter 21.

### Cohort overview and definitions

A retrospective cohort study design was utilized. All HIV‐positive adults (≥ 18 years old) recorded in Tier.net 20 as having started ART at the three study clinics between 1 January 2014 and the end of November 2015 were screened for inclusion. Individuals were excluded from the analysis if they did not have a recorded baseline CD4 count.

Baseline CD4 count was defined as the most recent CD4 count recorded in the 6 months prior to starting ART. Baseline eGFR was estimated from the most recent serum creatinine (SCR) measurement recorded in the 6 months prior to starting ART. For this analysis, American National Kidney Foundation criteria were used to define levels of renal dysfunction, that is, mild (eGFR 60–89 mL/min), moderate (eGFR 30–59 mL/min) or severe (eGFR < 30 mL/min), to best align our data with other published data 22. The moderate and severe renal dysfunction categories were combined (eGFR < 60 mL/min) for analysis of both baseline renal dysfunction (eGFR < 60 mL/min) and incident renal dysfunction (eGFR < 60 mL/min). The definition of incident eGFR < 60 mL/min was based on one recorded eGFR < 60 mL/min. Baseline tuberculosis (TB) was defined as being on TB treatment at the time of ART start.

Attrition was defined as being 3 months late for a scheduled clinic appointment and included all individuals no longer in care as a result of loss to follow‐up (LTFU) or death. Death and LTFU were not analysed separately in this study, as death was not consistently recorded in Tier.net. Transfer out (TFO) was defined as elective transfer of an individual to another ART clinic documented in Tier.net.

**Figure 1:**
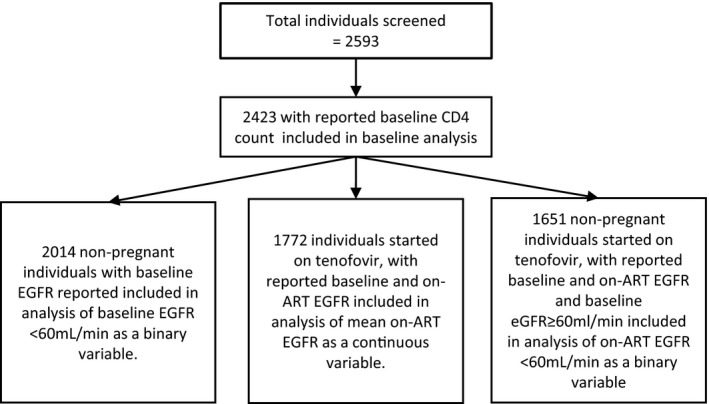
Overview of individuals included in the respective analyses.

All individuals were followed up for a minimum of 6 months until the end of May 2016 (the date of administrative censor) or until the date of attrition or TFO if that occurred earlier. For the analysis of incident renal dysfunction, as a binary variable, individuals were censored after the first episode of incident renal dysfunction (eGFR < 60 mL/min). All analyses of eGFR after ART initiation (on ART eGFR) were restricted to individuals starting a TDF‐containing ART regimen (Fig. 1). Individuals with baseline TB were included in analysis of on‐ART eGFR.

### Data sources and management

Routine DOH and NHLS data were used in this study. Unless otherwise stated, all data were extracted from Tier.net 20. Data for SCR results were extracted from the NHLS database and linked to Tier.net data using the DOH unique identifier. eGFR was calculated from SCR, age and sex using the Modification of Diet in Renal Disease Study (MDRD) Study equation [eGFR = 175 × standardized SCR^−1.154^ × age^−0.203^ × 1.212 (if black) × 0.742 (if female)] 23, with omission of the racial category factor. This method for estimating eGFR from serum creatinine has been previously validated in the South African context and shown to be more accurate than the Chronic Kidney Disease Epidemiology Collaboration (CKD‐EPI) equations 16, 24, 25. MDRD is the method of choice for estimating eGFR from results of the current serum creatinine assay used by the NHLS in South Africa and at the clinics included in this study 21, 26. A sensitivity analysis using eGFR calculated with the CKD‐EPI equation, without the race factor, as recommended by Seape *et al*. 25, was also completed for this study.

Creatinine data missing from the NHLS data extracts were, where available, extracted from clinic folders. Data from the DOH electronic TB monitoring system, ETR.net, were used to identify individuals with baseline TB. All data fields and data linkages were reviewed for completeness and accuracy.

### Analysis

Standard descriptive analyses including absolute numbers and proportions were used to describe baseline characteristics. The similarity of baseline characteristics across baseline CD4 count categories was analysed using χ^2^ and Kruskal–Wallis tests. Logistic regression was used to model the association of baseline characteristics with baseline renal dysfunction. Mixed effects linear regression was performed using a model with random intercept at the participant level with random slope for time from starting ART to eGFR measurement. Time‐to‐event analyses for incident eGFR < 60 mL/min as a binary variable were performed using Kaplan–Meier survival estimates in nonpregnant individuals with baseline eGFR ≥ 60 mL/min. Cox regression was used in analysis of baseline characteristics associated with hazard of eGFR < 60 mL/min on ART. This analysis was restricted to nonpregnant individuals and those with baseline eGFR ≥ 60 mL/min. Women pregnant at the time of starting ART were excluded from the analysis of baseline on‐ART eGFR < 60 mL/min in view of increases in eGFR levels during pregnancy of up to 50% 27. Within this group of participants, a sensitivity analysis using eGFR calculated with the CKD‐EPI equation without the race factor was performed.

The same baseline characteristics, namely age, sex, CD4 count, eGFR, TB status, clinic where treatment was received, year in which ART started, pregnancy at baseline and previous ART exposure of greater than 3 months duration were included in all multivariate models unless otherwise stated. Baseline characteristics for inclusion in multivariate modelling were selected primarily on the basis of clinical significance and data availability. Likelihood ratio tests were used to assess the regression model goodness of fit. All the multivariate models showed a good fit (*P* < 0.05). For Cox regression, Schoenfeld residuals were used to test the proportional hazard assumptions. The reference category for categorical variables was selected by using the category with the largest sample size or based on clinical significance. Comparison of baseline characteristics between individuals excluded from analyses because of missing eGFR results and those retained in analyses was performed using logistic regression. Logistic regression was also used to compare baseline characteristics of individuals retained in the study cohort and those experiencing attrition or TFO. Analyses were performed using stata version 14 (StataCorp LP, College Station, TX).

### Ethics

The HPTN 071 (PopART) trial has been approved by the Stellenbosch University Health Research Ethics Committee (SU HREC) (Ref. No. N12/11/074) and the London School of Hygiene and Tropical Medicine Research Ethics Committee (Ref. no. 6326). All HIV‐positive individuals starting ART outside of provincial ART guidelines provided informed consent. Additional SU HREC approval for the use of individual‐level DOH data in this study, including a waiver for informed consent, was received from SU HREC (Ref. no. N12/11/074A) as well as from the Western Cape Government (Ref. no. WC_2015RP51_715) and City of Cape Town (Ref. no 10529) research committees.

## Results

### Baseline characteristics

Overall, 2593 individuals were screened for study inclusion; 170 (6.6%) were excluded because of missing baseline CD4 cell counts, leaving 2423 (93.4%) individuals included in the baseline analysis (Fig. 1 and Table 1). The median baseline CD4 count was 328 cells/μL [interquartile range (IQR) 195–468 cells/μL]. The number of individuals in the baseline CD4 cell categories varied between 631 (26.0%) for CD4 counts of 0–200 cells/μL and 502 (20.7%) for CD4 counts > 500 cells/μL. The median age was 31 years (IQR 26–38 years), and the majority of participants (1643; 67.8%) were women. Baseline eGFR was available for 2141 of 2423 (88.4%) individuals. The median baseline eGFR was 110.5 mL/min (IQR 93.5–131.9 mL/min). A total of 47 (1.9%), 394 (16.3%) and 1700 (70.2%) individuals had baseline eGFR < 60, 60–89 and ≥ 90 mL/min, respectively.

**Table 1:** Baseline characteristics

	CD4 count at baseline					
	0–200 cells/μL	201–350 cells/μL	351–500 cells/μL	> 500 cells/μL	Total	*P* value
Baseline CD4 category [*n* (%)]	631 (26.0)	708 (29.2)	582 (24.0)	502 (20.7)	2423	
Age (years)						
[median (IQR)]	33 (29.0–40.0)	31 (25.0–37.0)	31 (26.0–37.0)	30 (25.0–37.0)	31 (26.0–38.0)	< 0.001
Age category [*n* (%)]						
18–25 years	79 (12.5)	179 (25.3)	142 (24.4)	134 (26.7)	534 (22.0)	< 0.001
26–35 years	312 (49.6)	311 (43.9)	272 (46.7)	227 (45.2)	1122 (46.3)	
36–45 years	167 (26.5)	138 (19.5)	109 (18.7)	87 (17.3)	501 (20.7)	
46–55 years	57 (9.0)	63 (8.9)	46 (7.9)	44 (8.8)	210 (8.7)	
> 55 years	17 (2.7)	17 (2.4)	13 (2.2)	10 (2.0)	57 (2.3)	
Gender [*n* (%)]						
Female	355 (56.3)	463 (65.4)	421 (72.3)	404 (80.5)	1643 (67.8)	< 0.001
Male	276 (43.7)	245 (34.6)	161 (27.7)	98 (19.5)	780 (32.1)	
Pregnant at ART start [*n* (%)]	14 (3.9)	39 (8.4)	41 (9.7)	48 (11.9)	142 (8.6)	< 0.001
Baseline TB [*n* (%)]	162 (25.7)	56 (7.9)	41 (6.7)	26 (5.2)	285 (11.8)	< 0.001
Clinic [*n* (%)]						
Metro 1	88 (13.9)	113 (15.9)	126 (21.7)	127 (25.3)	454 (18.7)	< 0.001
Metro 2	299 (47.4)	301 (42.5)	231 (39.7)	191 (38.1)	1022 (42.2)	
Rural	244 (38.7)	294 (41.5)	225 (38.7)	184 (36.7)	947 (39.1)	
Previous ART > 3 months [*n* (%)]	27 (4.3)	10 (1.4)	7 (1.2)	5 (1.0)	49 (2.0)	0.005
ART start year [*n* (%)]						
2014	161 (25.5)	208 (29.4)	160 (27.5)	161 (32.1)	690 (28.5)	0.091
2015	470 (74.2)	500 (70.6)	422 (72.5)	341 (67.9)	1733 (71.5)	
Baseline eGFR (mL/min)						
Median (IQR)	106.7 (89.5–130.6)	110.6 (94.3–129.7)	111.1 (93.1–131.7)	112.9 (96.5–134.1)	110.5 (93.5–131.9)	0.0186
< 60 mL/min [*n* (%)]	24 (3.8)	14 (1.9)	4 (0.7)	5 (1.0)	47 (1.9)	< 0.001
60–89 mL/min [*n* (%)]	114 (18.1)	106 (14.9)	100 (17.2)	74 (14.7)	394 (16.3)	
≥ 90 mL/min [*n* (%)]	399 (63.3)	506 (71.4)	418 (71.8)	377 (75.1)	1700 (70.2)	
Missing [*n* (%)]	94 (14.8)	82 (11.7)	60 (10.3)	46 (9.2)	282 (11.6)	

This table includes all 2423 participants included in the study sample. χ^2^ and Kruskal–Wallis tests were used to measure the heterogeneity of baseline characteristics across the baseline CD4 count categories. Overall, there was significant heterogeneity across baseline CD4 count categories. American Kidney Foundation definitions were used for defining renal function using estimated glomerular filtration rate (eGFR). Moderate and severe renal dysfunction was defined as eGFR < 60 mL/min; mild renal dysfunction was defined as eGFR = 60–89 mL/min; normal renal function was defined as eGFR ≥ 90 mL/min.

ART, antiretroviral therapy; IQR, interquartile range; TB, tuberculosis.

### Baseline factors associated with baseline eGFR < 60 mL/min

Univariate and multivariate logistic regression analyses of the association between baseline characteristics and baseline eGFR < 60 mL/min among 2014 nonpregnant individuals (Fig. 1) with a reported baseline CD4 count are shown in Table 2. In this subset of individuals, 47 of 2014 (2.3%) had a baseline eGFR < 60 mL/min. The adjusted odds ratios (aORs) of baseline eGFR < 60 mL/min across baseline CD4 count categories were 0.29 [95% confidence interval (CI) 0.11–0.80] for CD4 counts > 500 cells/μL, 0.22 (95% CI 0.08–0.59) for CD4 counts of 351–500 cells/μL and 0.48 (95% CI 0.24–0.97) for CD4 counts of 201–350 cells/μL compared with CD4 counts of 0–200 cells/μL. Baseline eGFR < 60 mL/min was also more common in individuals aged > 35 years, with aORs of 3.38 (95% CI 1.64–6.95) for ages 36–45 years and 6.40 (95% CI 3.06–13.37) for ages > 45 years compared with individuals aged ≤ 35 years. Baseline eGFR < 60 mL/min was more common among men (aOR 1.99; 95% CI 1.08–3.66) than women. There were no other baseline variables associated with baseline eGFR < 60 mL/min.

**Table 2:** Baseline factors associated with baseline renal dysfunction

	Baseline factor	Number of events/number of eligible individuals (%)	Crude odds ratio (95% CI)	*P* value	Adjusted odds ratio (95% CI)	*P* value
Baseline CD4 count (cells/μL)	> 500	5/409 (1.2)	0.19 (0.07, 0.56)	< 0.001	0.29 (0.11, 0.8)	0.003
	351–500	4/486 (1.0)	0.17 (0.06, 0.47)		0.22 (0.08, 0.59)	
	201–350	14/596 (2.4)	0.38 (0.18, 0.83)		0.48 (0.24, 0.97)	
	0–200	25/523 (4.8)	1		1	
Gender	Male	29/670 (4.3)	2.99 (1.68, 5.34)	< 0.001	1.99 (1.08, 3.66)	0.027
	Female	20/1344 (1.5)	1		1	
Age at ART start (years)	18–35	14/1347 (1.0)	1	< 0.001	1	< 0.001
	36–45	18/431 (4.2)	4.14 (2.04, 8.41)		3.38 (1.64, 6.95)	
	> 45	17/236 (7.2)	7.39 (3.59, 15.21)		6.40 (3.06, 13.37)	
Clinic	Metro 1	15/852 (1.8)	1	0.233	1	0.186
	Metro 2	23/786 (2.9)	1.68 (0.87, 3.25)		1.96 (0.93, 4.11)	
	Rural	11/376 (2.9)	1.68 (0.76, 3.69)		1.84 (0.77, 4.39)	
Baseline TB	TB	8/237 (3.4)	1.55 (0.84, 2.88)	0.175	0.97 (0.5, 1.87)	0.920
Previous ART > 3 months	Yes	1/40 (2.5)	1.03 (0.14, 7.64)	0.978	0.86 (0.11, 6.68)	0.889
Year of ART start	2014	15/584 (2.6)	0.41 (0.37, 0.45)	0.801	0.78 (0.38, 1.56)	0.489
	2015	34/1430 (2.4)	1		1	

The table includes a total of 2014 nonpregnant individuals with recorded baseline CD4 count and baseline estimated glomerular filtration rate (eGFR). Women who were pregnant at baseline were excluded because of changes in eGFR associated with pregnancy. Age categories 18–25 and 26–35 years were combined and age categories 46–55 and > 55 years were combined for the regression analysis because of small numbers of events in some age groups. *P* values were calculated using likelihood ratios.

ART, antiretroviral therapy; CI, confidence interval; TB, tuberculosis.

### Baseline factors associated with on‐ART eGFR

A total of 651 (26.9%) of the 2423 individuals included in the baseline analysis were excluded from the analysis of mean eGFR after ART initiation for the following reasons: (1) missing baseline eGFR (282 individuals; 43.3%); (2) started on an ART regimen that did not contain TDF or unknown baseline ART regimen (53 individuals; 8.1%) and (3) no reported eGFR after ART initiation (on‐ART eGFR) (316 individuals; 48.5%). The remaining 1772 (73.1%) individuals were included in the analysis of on‐ART eGFR after ART initiation and were followed up for a median of 12.1 (IQR 7.9–16.9) months, during which time 329 (18.6%) experienced attrition and 85 (4.8%) experienced TFO.

The mean on‐ART eGFR was 114.6 mL/min (95% CI 113.6–115.6 mL/min). Multivariate mixed effects linear regression showed that, compared with those with baseline eGFR ≥ 90 mL/min, mean on‐ART eGFR was 37.49 mL/min (95% CI 28.52–46.46 mL/min) lower among individuals with baseline eGFR < 60 mL/min and 22.47 mL/min (95% CI 19.34–25.60 mL/min) lower among individuals with baseline eGFR 60–89 mL/min (Table 3). Mean on‐ART eGFR was 20.56 mL/min (95% CI 15.57–25.55 mL/min) greater among women pregnant at baseline and 6.80 mL/min (95% CI 3.67–9.92 mL/min) lower among individuals attending the Metro 2 clinic compared with Metro 1. There was no difference in mean eGFR when comparing individuals treated at the Rural clinic and Metro 1. There was a mean change in on‐ART eGFR of −0.38 mL/min (95% CI −0.53 to −0.22 mL/min) per month of follow‐up.

**Table 3:** Baseline factors associated with on‐antiretroviral therapy (ART) estimated glomerular filtration rate (eGFR)

Estimated eGFR	Crude coefficient (95% CI)		Adjusted coefficient (95% CI)	
Baseline CD4 count				
> 500 cells/μL	2.72 (−1.3, 6.74)	0.552	−0.79 (−4.38, 2.8)	0.974
350–500 cells/μL	1.94 (−1.93, 5.81)		−0.13 (−3.56, 3.29)	
200–350 cells/μL	1.28 (−2.4, 4.97)		−0.17 (−3.42, 3.08)	
0–200 cells/μL	1		1	
Baseline eGFR				
< 60 mL/min	−27.08 (−30.26, −23.91)	< 0.001	−37.49 (−46.46, −28.52)	< 0.001
60–89 mL/min	−44.46 (−53.74, −5.17)		−22.47 (−25.60, −19.34)	
≥ 90 mL/min	1		1	
Gender				
Male	−4.65 (−7.57, −1.73)	0.002	1.27 (−1.42, 3.96)	0.356
Female	1		1	
Age category				
18–35 years	1	< 0.001	1	< 0.001
36–45 years	−14.89 (−18.16, −11.61)		−10.3 (−13.36, −7.25)	
> 45 years	−22.35 (−26.48, −18.22) 7)		−13.3 (−17.22, −9.38)	
Pregnant at baseline				
Yes	25.99 (20.51, 31.47)	< 0.001	20.56 (15.57, 25.55)	< 0.001
Clinic				
Metro 1	1	< 0.001	1	< 0.001
Metro 2	−7.43 (−10.45, −4.4)		−6.80 (−9.92, −3.67)	
Rural	−2.15 (−5.77, 1.48)		−2.09 (−5.69, 1.52)	
Baseline TB				
Yes	1.67 (−1.63, 4.97)	0.382	−6.71 (−17.38, 3.96)	0.106
Previous ART of > 3 months				
Yes	−10.44 (−22.63, 1.75)	0.093	2.45 (−0.52, 5.42)	0.218
Year of ART start				
2014	−1.06 (−3.96, 1.83)	0.471	1.67 (−1.35, 4.68)	0.279
2015	1		1	
Time on ART				
Change/month	−0.36 (−0.51, −0.21)	< 0.001	−0.38 (−0.53, −0.22)	< 0.001

Modelling included 1772 individuals with reported baseline and on‐ART estimated glomerular filtration rate (eGFR), including pregnant women. The individual unique identifier was used as the panel variable. For categorical variables, the coefficient represents the difference in mean eGFR in mL/min from baseline compared with the reference category.

CI, confidence interval; TB, tuberculosis.

### Baseline factors associated with on‐ART incident eGFR < 60 mL/min

**Figure 2:**
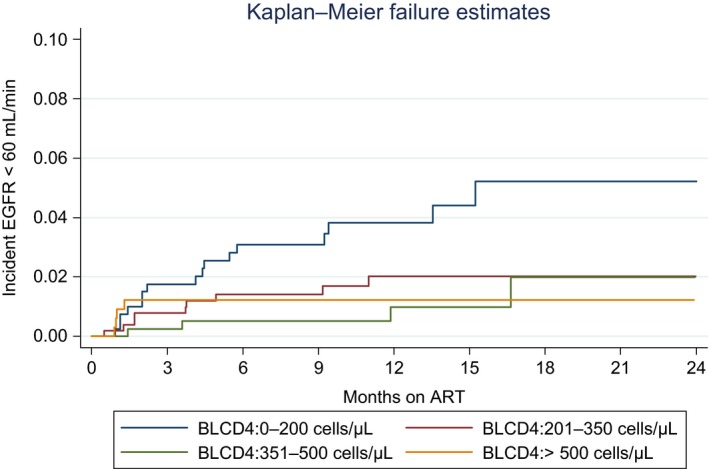
Kaplan–Meier estimates of estimated glomerular filtration rate (eGFR) < 60 mL/min on antiretroviral therapy (ART) in participants with baseline eGFR ≥ 60 mL/min. These participants included 1634 individuals with baseline eGFR ≥ 60 mL/min. Log‐rank test for difference in Kaplan Meier estimates by CD4 category: *P* = 0.011.

For analysis of incident eGFR < 60 mL/min, 106 women who were pregnant at baseline were excluded, leaving a sample size of 1665. Kaplan–Meier estimates showed higher rates of on‐ART eGFR < 60 mL/min over time at lower baseline CD4 counts (*P* = 0.011) (Fig. 2). There were 33 incident cases of eGFR < 60 mL/min occurring in 1665 individuals during 1750 person‐years (PY), giving an incidence rate (IR) of 1.9/100 PY (95% CI 1.3–2.6/100 PY) (Table 4). The IR of eGFR < 60 mL/min was highest during the first 6 months of ART (IR 2.3/100 PY; 95% CI 1.99–2.66/100 PY) compared with longer durations of ART; however, this difference was not statistically significant. The analysis restricted to 1634 nonpregnant individuals with baseline eGFR ≥ 60 mL/min showed that there were 27 incident cases of eGFR < 60 mL/min during 1722 person‐years (PY) of follow‐up (IR 1.6/100 PY; 95% CI 1.1–2.3/100 PY) (Table 4).

**Table 4:** On‐antiretroviral therapy (ART) incident estimated glomerular filtration rate (eGFR) < 60 mL/min by baseline eGFR category

	Baseline CD4 count (cells/μL)	All baseline eGFR		Baseline eGFR ≥ 60 mL/min		Baseline eGFR ≥ 90 mL/min	
		No. of events/person time	IR/100 PY	No. of events/person time	IR/100 PY	No. of events/person time	IR/100 PY
Sample size			1665		1634		1320
IR	0–200	16/418	3.8 (2.3, 6.2)	13/409	3.2 (1.8–5.5)	8/320	2.5 (1.2, 4.9)
	201–350	9/550	1.6 (0.9, 3.1)	6/538	1.1 (0.5–2.5)	4/443	0.9 (0.3, 2.4)
	301–500	4/425	0.9 (0.4, 2.5)	4/423	0.9 (0.4–2.5)	3/343	0.9 (0.3, 2.7)
	> 500	4/357	1.1 (0.4, 2.9)	4/353	1.1 (0.4–3)	0/295	0 (0, 0)
	All	33/1750	1.9 (1.3, 2.6)	27/1722	1.6 (1.1–2.3)	15/1401	1.1 (0.6, 1.8)

An event is defined as eGFR < 60 mL/min. Baseline eGFR cut‐offs were selected to align to American Kidney Foundation definitions; eGFR ≥ 60 mL/min was considered to represent normal eGFR or mild renal impairment and eGFR ≥ 90 mL/min normal renal function.

PY, person‐years; IR, incidence rate.

All individuals with recorded incident on‐ART eGFR had multiple on‐ART eGFR laboratory results. However, among the 27 individuals who had an incident eGFR < 60 mL/min in the subset of individuals included in multivariate analysis, only five (18.5%) had a further result that showed an eGFR < 60 mL/min recorded after their initial eGFR < 60 mL/min result. Of these five, one changed to a non‐TDF‐containing ART regimen and four remained on TDF. Four of 5 (80%) of these individuals, including the one that changed ART regimen, had a last recorded eGFR < 60 mL/min. Eight of the 27 (29.6%) had no further recorded eGFR after one episode of eGFR < 60 mL/min. In the remaining 14 cases, there was only one recorded eGFR of < 60 mL/min, with at least one subsequent eGFR > 60 mL/min recorded. Thirteen of these 14 (92.9%) individuals remained on TDF during the follow‐up period.

When the analysis was further restricted to 1320 nonpregnant individuals with baseline eGFR ≥ 90 mL/min, there were only 15 incident cases of eGFR < 60 mL/min (IR 1.1/100 PY; 95% CI 0.6–1.8/100 PY). Notably, when the analysis was limited to this group with normal baseline eGFR, there were no incident cases of eGFR < 60 mL/min among individuals with baseline CD4 counts > 500 cells/μL.

Multivariate Cox regression, which included 27 incident cases of eGFR < 60 mL/min among 1634 nonpregnant individuals with baseline eGFR ≥ 60 mL/min (Table 5), showed a lower hazard ratio of eGFR < 60 mL/min in higher baseline CD4 categories compared with baseline CD4 count < 200 cell/μL, but this finding was not statistically significant. There was a higher hazard ratio of incident eGFR < 60 mL/min among individuals with a baseline eGFR of 60–89 mL/min [adjusted hazard ratio (aHR) 2.62; 95% CI 1.17–5.83] compared with those with baseline eGFR ≥ 90 mL/min and among individuals aged > 45 years (aHR 3.25; 95% CI 1.30–8.14) compared with individuals aged ≤ 35 years. No other baseline characteristics were associated with incident eGFR < 60 mL/min.

**Table 5:** Baseline factors associated with on‐antiretroviral therapy (ART) incident estimated glomerular filtration rate (eGFR) < 60 mL/min among individuals with baseline eGFR ≥ 60 mL/min

	Crude hazard ratio (95% CI)	*P* value	Adjusted hazard ratio (95% CI)	*P* value
Baseline CD4 count				
> 500 cells/μL	0.36 (0.12, 1.11)	0.056	0.41 (0.13, 1.3)	0.131
351–500 cells/μL	0.3 (0.1, 0.92)		0.35 (0.11, 1.1)	
201–350 cells/μL	0.35 (0.13, 0.93)		0.38 (0.14, 1.03)	
0–200 cells/μL	1		1	
Baseline eGFR				
60–89 mL/min	3.46 (1.62, 7.39)	0.002	2.62 (1.17, 5.83)	0.019
≥ 90 mL/min	1		1	
Gender				
Male	1.46 (0.68, 3.15)	0.339	1.08 (0.48, 2.42)	0.845
Female	1		1	
Age category				
18–35 years	1	0.008	1	0.049
36–45 years	1.63 (0.61, 4.35)		1.24 (0.46, 3.39)	
> 45 years	4.33 (1.83, 10.29)		3.25 (1.30, 8.14)	
Clinic				
Metro 1	1	0.854	1	0.805
Metro 2	0.86 (0.37, 2.01)		0.74 (0.26, 2.12)	
Rural	0.74 (0.25, 2.17)		0.68 (0.19, 2.39)	
Baseline TB				
Yes	1.61 (0.7, 3.67)	0.261	1.27 (0.54, 3.04)	0.583
Previous ART of > 3 months				
Yes	3.2 (0.43, 23.61)	0.253	1.77 (0.23, 13.77)	0.588
Year of ART start				
2014	0.93 (0.41, 2.12)	0.868	1.11 (0.39, 3.18)	0.843
2015	1		1	

Nonpregnant Individuals with baseline eGFR < 60 mL/min were excluded from the analysis, and the model includes 1634 individuals with reported baseline eGFR ≥ 60 mL/min. Women who were pregnant at baseline were excluded because of changes in eGFR associated with pregnancy. The baseline category with the largest sample size was used as the reference. There were no incident eGFR < 60 mL/min cases among individuals reported to be pregnant at baseline; therefore, this variable was omitted from regression analysis. Age categories 46–55 and > 55 years were combined as > 45 years because of the limited sample size.

CI, confidence interval; TB, tuberculosis.

### Sensitivity analysis using the CKD‐EPI formula

When eGFR calculated with the CKD‐EPI equation was used, the mean on‐ART eGFR was 118.1 mL/min (95% CI 117.4–118.8 mL/min) and the number of incident eGFR < 60 mL/min cases reduced from 27 to 17 (IR 0.88/100 PY; 95% CI 0.06–0.14/100 PY). Multivariate analysis also did not show an association between baseline CD4 count or any other baseline factors and incident eGFR < 60 mL/min calculated using the CKD‐EPI equation.

### Analysis of missing data

Logistic regression using the full model showed increased rates of missing baseline eGFR results at higher baseline CD4 counts > 500 cells/μL (aOR 1.54; 95% CI 1.04–2.26) compared with baseline CD4 counts ≤ 200 cells/μL. Individuals with missing baseline eGFR results were less likely to be male (aOR 0.71; 95% CI 0.55–0.93) and more likely to attend the Rural clinic (aOR 1.54; 95% CI 10.01–2.33) rather than Metro 1. Individuals excluded from follow‐up analysis because of missing on‐ART eGFR were more likely to have a baseline CD4 count of 201–350 cells/μL (aOR 1.35; 95% CI 1.00–1.83). Individuals experiencing attrition or TFO were less likely to have started ART in 2015 (aOR 0.60; 95% CI 0.46–0.79) as opposed to 2014; less likely to be aged 36–45 years (aOR 0.72; 95% CI 0.55–0.97) or > 45 years (aOR 0.49; 95% CI 0.33 0.72) as opposed to ≤ 35 years and more likely to be treated at the Metro 2 clinic (aOR 1.46; 95% CI 1.09–1.95) rather than at Metro 1.

## Discussion

This is one of the first studies to evaluate the association between baseline CD4 count and renal dysfunction in the context of routine provision of ART regardless of CD4 count in a high‐burden programmatic setting. Probably as a result of this expanded ART access, the baseline median CD4 count in this study (328 cells/μL) was higher than that reported in previous studies from high‐burden settings 13, 16, 28. This study found a low baseline prevalence rate of eGFR < 60 mL/min (1.9%), similar to that in a large published study from Zambia 29. Also similar to previous studies 13, 16, 28, this study showed lower baseline prevalence of renal dysfunction at baseline CD4 counts > 200 cells/μL and among younger individuals.

Similar to other studies, this study showed a small mean decline in eGFR over time on tenofovir 16. The overall incidence rate of eGFR < 60 mL/min (1.9 cases/100 PY) was lower than that reported in a previous study (5.9 cases/100 PY) with a lower baseline median CD4 count that reported on the same endpoint 16. Also in contrast to previous studies, this study did not find an association between baseline CD4 count and average eGFR or renal dysfunction after ART initiation after adjustment for key baseline characteristics 10, 17. Notably, analysis of incident eGFR < 60 mL/min – restricted to individuals with a normal baseline renal function – showed a low incidence rate of eGFR < 60 mL/min of 1.1 cases/100 PY (95% CI 0.6–1.8 cases/100 PY) and no incident cases among individuals with baseline CD4 counts > 500 cells/μL. As in previous studies, this study showed baseline renal dysfunction to be a strong predictor of on‐ART renal dysfunction 13, 16, 29. The number of individuals who changed from a TDF‐containing regimen was small, and the data showed good recovery of renal function in the majority of individuals experiencing eGFR < 60 mL/min on ART.

Monitoring of renal function in high‐burden settings is challenging 13, 30. TDF continues to be widely used for first‐line ART in high‐burden regions, and in the absence of an affordable alternative, strategies to improve eGFR monitoring in individuals on TDF are needed 8, 21 Tenofovir alafenamide (TAF) is a tenofovir prodrug, with reported lower risk of renal toxicity 31, 32, 33. However, TAF interacts with rifampicin, which is used for TB treatment, which makes the introduction of this drug in resource‐limited settings with a high burden of TB challenging 9, 34, 35. Management of renal function on ART is further challenged by limited access to laboratory services in resource‐limited settings, and further research to evaluate the potential of more accessible point‐of‐care technology for monitoring renal function is needed 30. In settings where relevant laboratory services are available, nonadherence to eGFR monitoring is widely reported 30, 36.

This study used the MDRD equation without the race factor for calculation of the primary endpoint with a sensitivity analysis using the CKP‐EPI equation without the race factor. These analyses showed an overall mean on‐ART eGFR of 114 and 118 mL/min, respectively. The MDRD equation without the race factor is the current preferred method for calculating eGFR used by the NHLS in South Africa 26. Similar to previous studies, there were significant differences in the results when using these two equations. A previous study from South Africa showed cystatin C‐based estimations to be the most accurate for calculating eGFR 25. Of the creatinine‐based formulae, both MDRD and CKP‐EPI equations, both without the race factor, overestimated eGFR by 14.2 and 15.3%, respectively 25. Cystatin C‐based estimations are currently not affordable for routine use in many high‐burden settings 25.

These reported inaccuracies in the current methods commonly used for estimating eGFR are concerning and the strategy for eGFR monitoring in high‐burden settings needs revision. Models that use individual‐level factors such as age, sex, body mass index, diabetes status, blood pressure, serum creatinine and urine protein to identify individuals at high risk of renal dysfunction are used in some contexts 37. Published studies suggest that there is still a need to further develop and validate these ‘high‐risk’ models in high‐burden settings, where they might be successfully integrated in routine clinical care as part of a differentiated care approach 36, 37.

Despite a well‐established ART service in the Western Cape since 2004, and the provision of ART regardless of CD4 count in the PopART intervention since January 2014, a significant proportion (26%) of this cohort presented with baseline CD4 counts < 200 cells/μL. These data suggest that offering ART regardless of CD4 count, with increased numbers of individuals starting ART at higher CD4 counts, will have a positive impact on baseline renal function among individuals starting ART. Although these data did not show an association between baseline CD4 count and renal dysfunction in the primary analysis, the absence of incident renal dysfunction among individuals with baseline CD4 > 500 cells/μL in analysis restricted to individuals with normal baseline renal function is promising and should be further evaluated in larger studies.

### Strengths and weaknesses

This study presents high‐quality routine data from three clinics providing ART regardless of CD4 count, ahead of recent changes in WHO and national ART guidelines. The routine data used also benefited from data quality improvement interventions during the PopART intervention. The study has been a joint undertaking between researchers and clinical staff, ensuring that the analysis and interpretation of the data correctly reflect clinical activities. The study does, however, have a number of weaknesses that should be considered. Individual‐level factors, not recorded in the routine data, such as presence of diabetes and other comorbidities, may have confounded the study findings. In addition, although the three study clinics were similar in terms of structure and service delivery, health systems factors, which were not measured, may also have confounded study findings. Notably, among 27 individuals with incident eGFR < 60 mL/min, 29% did not have another eGFR recorded after a recorded eGFR < 60 mL/min, possibly as the result of the individuals being referred to another facility or hospital for ongoing care. There were significant amounts of missing data and attrition from ART care. Individuals excluded because of missing baseline eGFR were more likely to have higher baseline CD4 counts and inclusion of these individuals in the study sample could have resulted in lower rates of renal dysfunction being reported. There was also a limited sample size and follow‐up person time, which may have reduced the validity of several findings, such as the association between baseline CD4 count and incident eGFR < 60 mL/min, and the results of the sensitivity analysis. Follow‐up was censored at the first recorded event of eGFR < 60 mL/min; this precluded the study from reporting on subsequent additional episodes of eGFR < 60 mL/min in affected individuals, thereby potentially resulting in underreporting of the incidence of renal dysfunction after the first 6 months of ART. Comparison of both the prevalence of baseline eGFR < 60 mL/min and the incidence of eGFR < 60 mL/min after ART initiation between this study and other published studies was hampered by extensive heterogeneity of the definition of renal dysfunction across studies.

## Conclusions

Overall, this study showed low rates of renal dysfunction at baseline and on‐ART in a cohort of individuals started on TDF and suggests high durability for tenofovir as first‐line ART with a small decline in mean eGFR over time. The study showed a lower prevalence of baseline eGFR < 60 mL/min at higher CD4 counts and no association between baseline CD4 count and incident on‐ART eGFR < 60 mL/min. The study did, however, observe higher rates of incident renal dysfunction among older individuals and those with mild baseline dysfunction. Use of differentiated models of ART care, in which ‘high‐risk’ models are employed to identify individuals at high risk of renal dysfunction, may be an effective way to improve renal monitoring in high‐burden settings, and this should be the subject of ongoing research.

## Author contributions

PB, KN, GF, SF, and NB were responsible for the study concept. All authors contributed towards development of the manuscript and reviewed drafts, including the final draft.
